# Methodology paper: a novel phantom setup for commissioning of scanned ion beam delivery and TPS

**DOI:** 10.1186/s13014-019-1281-5

**Published:** 2019-05-09

**Authors:** O. Jäkel, B. Ackermann, S. Ecker, M. Ellerbrock, P. Heeg, K. Henkner, M. Winter

**Affiliations:** 1 Heidelberg Ion-Beam Therapy Center (HIT) at the University Hospital, Heidelberg, Germany; 2 Department of Medical Physics in Radiation Oncology, German Cancer Research Center (DKFZ), Heidelberg, Germany; 3 Heidelberg Institute for Radiation Oncology (HIRO), National Center for Radiation Research in Oncology (NCRO), Heidelberg, Germany

**Keywords:** Scanning ion beam, TPS commissioning, Phantoms

## Abstract

**Background:**

Commissioning of treatment planning systems (TPS) and beam delivery for scanned light ion beams is an important quality assurance task. This requires measurement of large sets of high quality dosimetric data in anthropomorphic phantoms to benchmark the TPS and dose delivery under realistic conditions.

**Method:**

A novel measurement setup is described, which allows for an efficient collection of a large set of accurate dose data in complex phantom geometries. This setup allows dose measurements based on a set of 24 small volume ionization chambers calibrated in dose to water and mounted in a holder, which can be freely positioned in a water phantom with various phantoms mounted in front of the water tank. The phantoms can be scanned in a CT and a CT-based treatment planning can be performed for a direct benchmark of the dose calculation algorithm in various situations.

**Results:**

The system has been used for acceptance testing in scanned light ion beam therapy at Heidelberg Ion Beam Therapy Center for scanned proton and carbon ion beams. It demonstrated to be useful to collect large amounts of high quality data for comparison with the TPS calculation using various phantom geometries.

**Conclusion:**

The setup is an efficient tool for commissioning and verification of treatment planning systems. It is especially suited for dynamic beam delivery, as many data points can be obtained during a single plan delivery, but can be adapted also for other dynamic therapies, like rotational IMRT.

## Background

The increasing complexity of modern precision radiotherapy requires dedicated quality assurance methods for all steps of the radiotherapy process. Especially for dynamic and high precision therapies, like scanned light ion beam therapy or rotational IMRT, it is important to assess the accuracy of dose calculation in the TPS and of the dose delivery itself. This requires experimental data taken under conditions of various complexity. While many systems allow the measurement of absorbed doses in homogenous phantoms, it is more difficult to measure doses in more complex situations including inhomogeneities or irregular geometries or a combination of both, as it is suggested in the recommendations of AAPM [1] and IAEA [2].

A typical scenario after configuring and during commissioning of a TPS, is thus to perform dosimetric measurements for e.g. a box shaped volume, delivered to a water tank to check the performance of the dose algorithm and treatment delivery in a situation, where it should allow for highly accurate dose calculation. In a next step, typically inhomogeneous phantoms are employed, usually starting with inhomogeneities in depth (large slabs of phantom material in various layers) to check the validity of the algorithm for correction of radiological path lengths. To investigate scatter corrections, lateral inhomogeneities are typically used. The first two situations can easily be solved, by using a water tank with or without combinations of slabs of phantom material in front and measuring dose with an ionization chamber in the water tank. Introduction of lateral inhomogeneities is usually investigated by using high resolution 2D detectors, like films [3] or a 2D ionization chamber array [4].

To simulate more realistic situations, like in a patient, anthropomorphic phantoms are used. The problem arising here, is that in these phantoms it is more difficult to accurately assess the dose distribution. Either point measurements are performed at fixed predefined positions in the phantom [3, 5] or 2D measurements are performed using CCDs or film detectors [3, 4], inserted at a fixed position in or behind the phantom. All three solutions have some limitations:

- Using single ionization chambers, leads to accurate dose determination, however, only very limited information on the overall dose distribution is obtained;
- Using CCD or film detectors leads to much more detailed information in two dimensions (still at a fixed position), but is more of qualitative nature, as film dosimetry is connected to larger uncertainties, esp. for light ion beams [6].
- 2D arrays of ion chambers provide typically lower accuracy, than a thimble type ionization chambers [6] and their spatial resolution is also limited. Moreover, thimble type chambers do provide a larger dose range, since recombination effects are small, even for scanned beams [6].

In this note we describe the methodology of a new setup, which allows to assess the dose distribution in a realistic situation with high accuracy and high resolution: the absorbed dose can be determined with a 1% precision reproducibility) and about 3% accuracy (connected to the dosimetry protocol) with a spatial resolution of below 1 mm.

## Methods

### Phantom construction and setup

To assess the accuracy of the dose algorithm of a pencil beam scanning system for proton and carbon ion beams at the Heidelberg Ion-Beam Therapy Center (HIT), we developed a phantom setup, which combines the advantage of the high accuracy of ionization chamber dosimetry with a flexible setup of various phantom materials, including an Alderson head phantom.

To allow for such a solution, we modified our standard setup for field-specifc dosimetric verification, which is described in [7]. The setup uses a commercial water tank (MP3-P, PTW, Freiburg, Germany) including a computer controlled 3D motion of a measuring device in water. The measuring device itself is a combination of 24 small ionization chambers (PTW Pinpoint chambers Type 31,015) mounted in a single PMMA holder, which allows for measurement of dose at 24 positions simultaneously and with varying position of the holder within the tank (no independent motion of the chamber is possible). The chambers are arranged in three rows of eight chambers each, where the rows are oriented laterally (left-right in beams-eye-view) and each row has a different vertical position (up-down in beams-eye-view), to avoid shadowing each other (when irradiated with a horizontal beam) and are calibrated in dose to water. The setup is shown in Fig. 2.

A set of mechanical adapters was designed to allow fixation of various phantoms directly at the entrance window of the water tank. Three types of phantoms can be mounted to the front window:

- a holder for fixation of slab phantoms either as a stack of several materials (laterally homogeneous) or including lateral inhomogeneity, by combining smaller slabs next to each other; the slabs can be mounted within or in front of the water tank;
- a holder for fixation of wedge shaped material (usually PMMA), to investigate non-perpendicular entrance of the beam to the phantom;
- a holder for fixation of an anthropomorphic head phantom. To avoid unnecessary air cavities, the phantom head was cut in a sagittal plane.

#### Slab phantoms

As phantom materials, tissue equivalent types of material of 1 cm thickness were used. In our case Gammex material (Sun Nuclear Inc., Middleton, Wisconsin) was chosen, as these materials are also the basis for CT calibration in our center. The large slabs were cut into smaller pieces of 10 × 10 cm^2^ (laterally homogeneous slabs) or 5x10cm^2^ in order to combine them to laterally inhomogeneous slabs. The smaller pieces can be clipped mechanically to the front of the water tank, by using a dedicated adapter. The adapter itself can be mounted to existing bores at the tank.

#### Wedge phantom

**Fig. 1:**
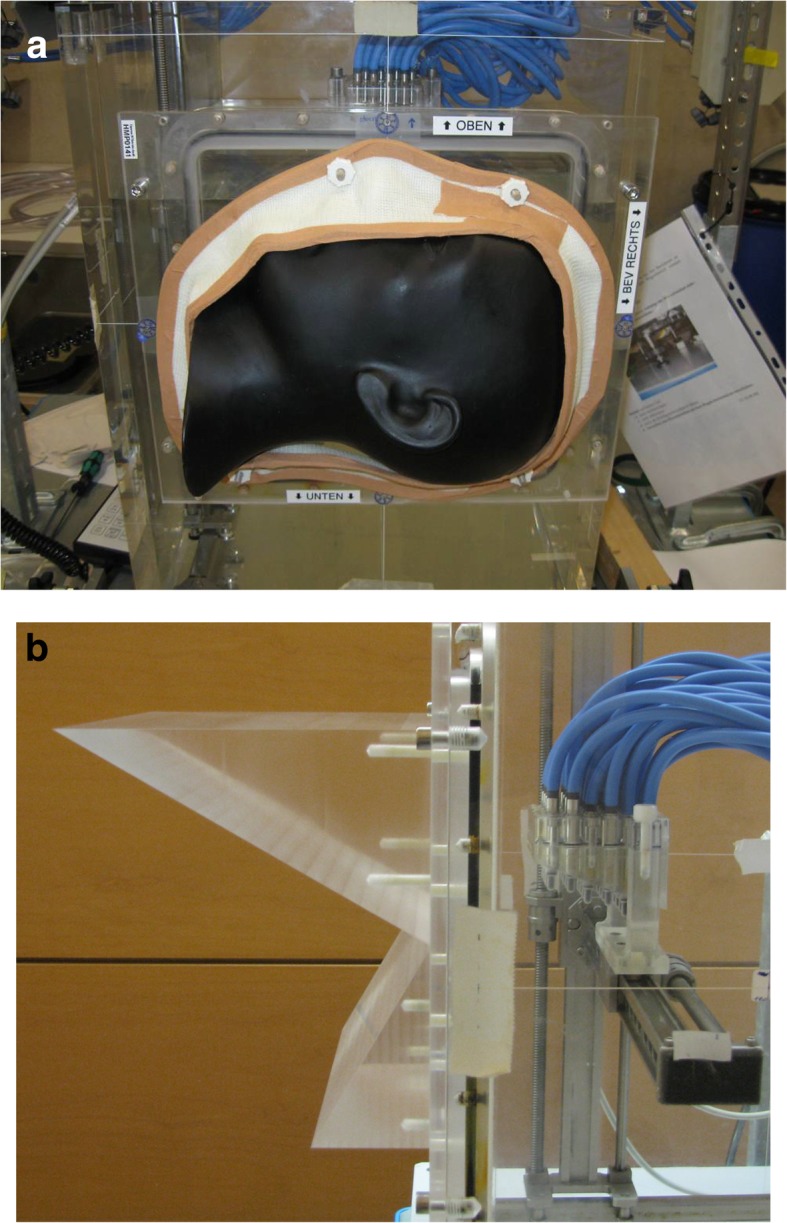
**a** The anthropomorphic head fixed with a thermoplastic mask to the entrance window of the water tank for irradiation with a horizontal beam. **b** Same setup for the PMMA double wedge phantom with a 30° (lower half) and 60° inclination (lower half). The block of 24 ionization chambers can be seen to the right of the wedge in water

In order to investigate situations with non-perpendicular alignment of beam and water tank, a wedge made of PMMA was machined, which has different angles on both sides: it was designed to exhibit an angle of 30° and 60°, respectively (see Fig. 1b). This wedge has a lateral size of 20 × 20 cm^2^ and can be mounted directly in the entrance window of the water tank. An additional double wedge has also been manufactured. The latter consists of two wedges with different angle (60° and a 30° angle), which comprise the two sides of the phantom with increasing thickness from the center to the outside.

#### Antrophomorphic phantom

As an anthropomorphic head phantom, the Alderson RANDO head phantom (Radiology support Devices, Long Beach, CA) was used. This phantom consists of a realistic bone structure, which mimics the bony anatomy of an adult skull. All the soft tissue is replaced by a dedicated polymer material, which also gives the phantom a complex outer shape of a human skull.

This phantom was cut in two halves along a sagittal plane in a mechanical workshop by the vendor according to our needs. To avoid an irregular surface of the cutting plane, this plane was coated with a thin layer like the outside of the head. A very smooth and hard surface of the mounting plane was resulting from this procedure. Since stable mechanical fixation of the head phantom to the water tank is not easily achievable, we decided to use a mask fixation similar to a system developed for patient fixation in our institute. A thermoplastic bandage material was molded to a PMMA frame, covering the outer part of the head phantom in such a way, that the head is thoroughly pressed against the tank window, when the adapter is mounted to the water tank.

### Treatment planning

#### Imaging data

In order to perform treatment planning for the various configurations, the density information of the phantom components needs to be included in the TPS. To achieve that, an artificial CT imaging data cube was created. This imaging cube consists first of density information for the water tank, i.e. including the water and a slab of material representing the PMMA wall of the entrance window. In addition, the density information of the materials is needed. For the tissue slabs and PMMA wedge, we parameterized their geometric shape and included this information directly in the artificial CT-cube.

For the Alderson head, we prepared a high resolution CT scan of the phantom, mounted to the CT table. For this purpose, a separate adapter was designed, which allows fixation of the head to the CT table. Imaging protocols were chosen identical to the standard patient imaging protocols used for routine treatment planning. The phantom, however, was mounted with CT slices being taken in sagittal plane of the head, in order to minimize partial volume artifacts at the surface of the phantom, which is mounted to the water tank. CT resolution was chosen as in our standard CT protocol for patients (1 mm resolution in-plane and 3 mm slice thickness). The resulting CT data of the head phantom were combined with a synthetic data cube of the water tank, to obtain a correct representation of the overall phantom setup, which can be imported into the treatment planning system.

#### Treatment planning system

The TPS used at HIT is the Siemens Syngo RT planning for particles (Siemens, Munich), which was installed initially in 2008 and was commissioned in 2009. During this commissioning process various treatment plans for the aforementioned phantom configurations were prepared, involving various regular geometric targets (cubes, rings, c-shaped volumes) at varying depth, but also more realistic patient-like plans for both proton and carbon ion beams.

The treatment plans were all based on the CT data, described above. The planning system features a QA function to support dosimetric verification of treatment plans. Using this function, various measurement positions can be prepared, while the position of the 24 ionization chambers within the dose distribution are visible. By doing so, interesting regions in the dose distribution can be selected. For patient plan verification, the displayed dose is the absorbed dose calculated in water for the specific phantom setup and based on the control file optimized for the RBE weighted dose distribution desired for the underlying patient plan. For QA purposes, the TPS also allows for direct optimization of absorbed dose distribution in a patient. The doses are calculated in the TPS at the effective point of measurement of the ionization chambers.

### Measurement and data analysis

The measurements are performed after aligning the phantom according to the wall-mounted lasers in the treatment room. The ionization chambers are read out by two multi-channel dosimeters with 12 channels each (PTW, Multidos). Readout of the dosimeters and positioning of the ion chamber array is remote controlled from the treatment control room by means of a TCP-IP connection. At HIT, this control software is completely integrated into the treatment control software (TCS).

The predefined measurement positions are then used to perform measurements of the dose at these positions using the specific setup of the phantoms chosen for planning. The measured data can then be stored and read into the control software again for direct analysis. An integrated procedure transfers the measured charge information from the TCS to the application system (RTTPT), converts it to dose and compares with the TPS prediction. Within this software a direct comparison of measured values and values calculated by the treatment planning system can be performed.

The measured and calculated dose can be displayed and analyzed according to a predefined protocol. In our case, the deviation for each point, as well as the mean and maximum deviations, calculated in the TPS, are recorded. In addition, the dose gradient at the point of measurement is recorded, allowing better interpretation of the results.

## Results

**Fig. 2:**
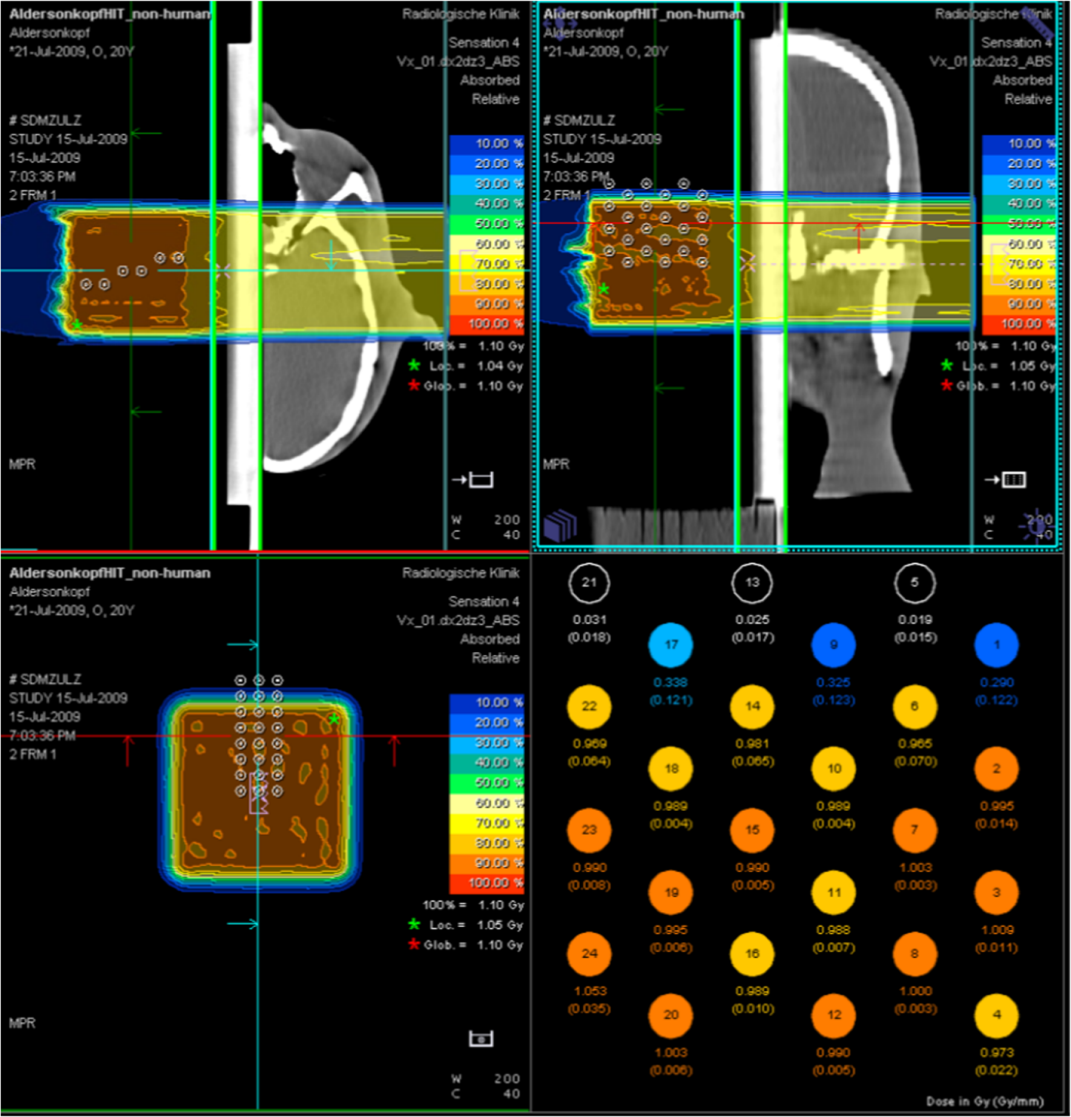
Screenshot from the Siemens Syngo TPS, showing the CT data of the head phantom together with the dose distribution for a carbon field in three sections (top left: from the side; top right from the top; bottom left: beam’s-eye-view). The measurement positions are displayed in each section as the white circles. The measured doses and dose gradients for the 24 ionization chambers are shown in the bottom right. Here, also the three rows (left to right) with eight chambers each (top-down) can be seen in one plane

**Fig. 3:**
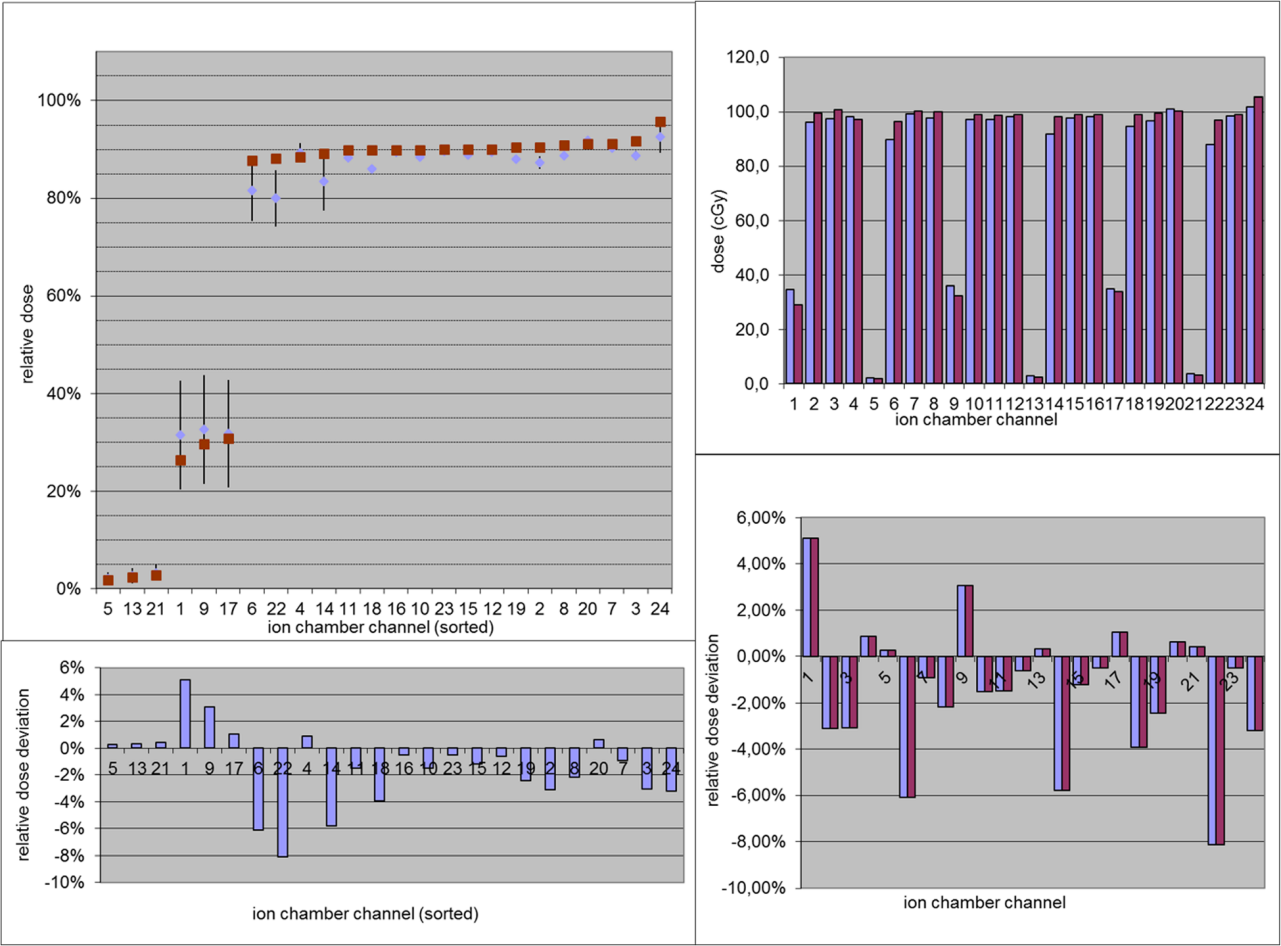
Screenshot from the analysis tool, showing the planned dose values in red and measured values in blue for the measurement position shown in Fig. 2 in a carbon field (top left: channels sorted according to relative dose; top right: absolute dose sorted by channel). In the plot, the error bars represent the dose gradient at the respective points. For both dose displays the deviations relative to the maximum are given in the bottom

We have established a system for dosimetric verification of the dose calculation algorithm for commissioning of treatment planning systems. The system was used extensively during the commissioning of the horizontal beam lines of the HIT facility and also proved to be very helpful and efficient during commissioning of the gantry. The system allows for a very efficient measurement and analysis of a large amount of data. As an example, during commissioning, measurements for a given phantom and plan were repeated typically 15times, resulting in 360 data points, which could be obtained in less than one hour. Moreover, the system allows to measure absorbed dose rather than relative dose, which is important for assessment of dose distributions in therapy with protons and ion beams. The dose can be measured with this setup with a precision of 1% and an accuracy of about 3% (connected to the dosimetry protocol) with a spatial resolution of below 1 mm. As compared to most existing solutions (and also our earlier system described in [7]), the combination of an inhomogeneous phantom with a water phantom allows also for a realistic assessment of the effects of range uncertainties e.g. behind an Alderson phantom, including among others also the uncertainties of the dose algorithm and beam delivery. s an example, the CT data and planned dose distribution for a box-shaped target behind the Alderson phantom (Fig. 1a), irradiated with a beam of carbon ions is shown in Fig. 2. The figure exhibits a screen shot from the TPS, where this information is combined. The figure shows the phantom and chamber position relative to the dose distribution in three planes and summarizes the measured dose for the 24 chambers. In total 15 different measurement positions were chosen for this test resulting in 360 data points. Figure 2 shows a position, where the chamber block is positioned laterally in the field, so that the lateral penumbra of the dose distribution is covered. The resulting planning data are compared to the measurements as shown in Fig. 3 for a single measurement. The distribution of all 360 data points, which were analyzed for this configuration, revealed that the mean deviation of measured vs. planned doses for carbon ions (protons) was − 1.5% (− 1%) with a standard deviation of 1.5% (2.5%). These numbers are important to asses to overall quality of calculated and delivered doses.

**Fig. 4:**
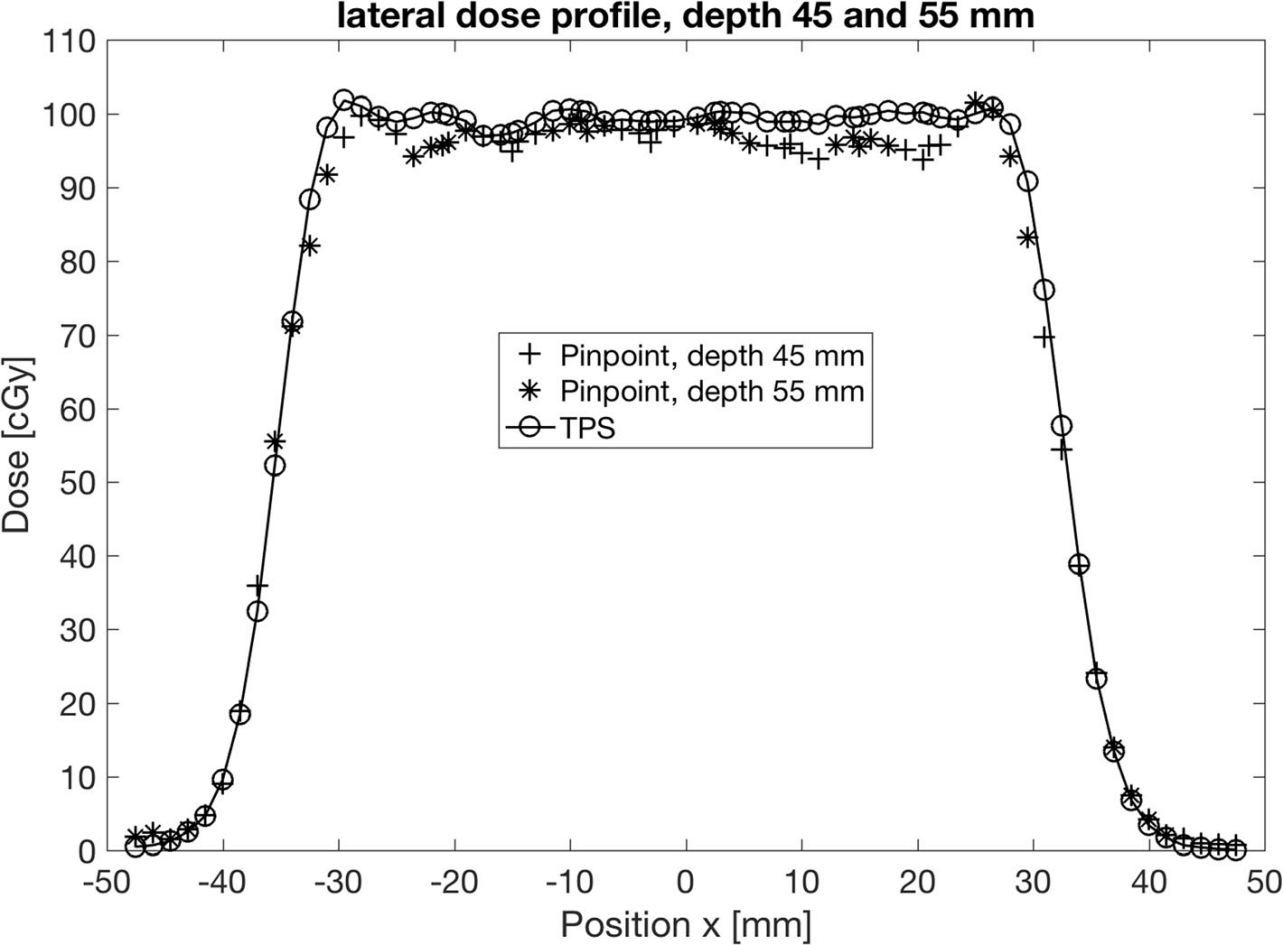
Lateral dose profile as measured with the Alderson phantom in a carbon field shown in Fig. 2. The data were derived from several repeated measurements at different lateral positions and for two different depths. The full line and open circles correspond to the planned lateral dose profile in the depth of 45 mm, the cross symbols are measured values at a depth of 45 mm (+) and 55 mm (*), respectively

In Fig. 4, another example of the high quality of data obtained with the presented phantom is shown. Here, a single lateral dose profile measured in the rectangular dose distribution shown in Fig. 2 is displayed. The data show, that the lateral fall-off and also the slight dose variations within the flat top are accurately reproduced by the TPS. For the profile, the data of several repeated measurements were compiled. Using the described setup, several such profiles can be obtained within short time.

## Discussion

The system we developed for dosimetric verification of a treatment planning algorithm for light ion beams largely relies on commercially available hardware. Only the adapters for the material slabs, wedge and head phantom to the water tank had to be produced in-house. This significantly reduces risks for failure of the system. The analysis software directly uses planning data exported from the TPS for the corresponding plan, but also allows for import of data from other systems, like e.g. a Monte Carlo algorithm. The measurement system allows for a 1 mm spatial resolution and 1% dose precision at 3% accuracy [6].

Currently, no equivalent system with similar accuracy and flexibility is described in the literature. Existing solutions rely mainly on film dosimetry in anthropomorphic phantoms [3, 5, 8–10], which does not allow a dose determination with an uncertainty below typically 10%, especially in the Bragg peak region. Other systems rely on single ionization chambers in a fixed position in a solid state phantom [5], which is, however, not very flexible and not suited to obtain a larger data set for benchmarking the TPS in a more complex situation. Moreover, the system allows to asses also the overall effects of range uncertainties in inhomogeneous phantoms resulting from the TPS and beam delivery.

In [10], a setup is described where a CT calibration phantom (model 062, CIRS, Inc. Norfolk VA) with several tissue equivalent discs is placed in front of a water tank. The chamber setup and read-out is similar to our setup and relies on the commercially available ionization chamber and the same read-out system as in [6]. It was used, to benchmark the density calibration of the TPS and not for more complex situations as described here. Moreover, it is not clear, if and how the phantom has been scanned in the CT and which parameters are used for this. Another publication [4] describes a system based on a 2D chamber array positioned below a water tank, where a bone slab is inserted. This is certainly reasonable for investigating the effects of scattering along the bone/water inhomogeneity, however, the system does not allow for more flexible setup, especially with a more complex phantom (like the Alderson head). Furthermore, the arrangement is using a fixed setup and it would not easily be possible to perform a measurement for a realistic treatment plan.

To underline the importance of accurate dosimetric measurements, we mention here Monte Carlo simulations, which are extensively used during the TPS commissioning in many facilities [3, 4, 8, 10, 11]. While the comparison of the Monte Carlo calculated data with the TPS calculated values alone is difficult to interpret, the measured data provide substantial additional information to allow for correct interpretation of the data. This allows for much more reliable conclusions on the accuracy of the treatment planning algorithms in the various situations.

Moreover the application of the phantom setup is not limited to scanned ion beams. It is certainly also useful for passive beam delivery and may also be adapted for dosimetric verification of dose distributions delivered with dynamic rotational Megavoltage X-ray therapy.

## Conclusion

We presented a flexible, novel system for dosimetric verification of treatment planning algorithms and beam delivery systems for dynamic beam delivery in precision radiotherapy, like scanned light ion beam therapy. The system largely consists of commercial products and allows for a very accurate and time efficient measurement of a large amount of data.

We believe such phantoms to be increasingly necessary in the future due to the increasing requirements in QA in radiotherapy. The described setup is especially suited for systematic measurements during acceptance testing and commissioning of a TPS and beam delivery system, using a variety of treatment plans and phantom geometries from simple to increasing complexity. These data are essential to understand the limitations of TPS algorithms.
